# Occurrence of autoantibodies against skin proteins in patients with hereditary epidermolysis bullosa predisposes to development of autoimmune blistering disease

**DOI:** 10.3389/fimmu.2022.945176

**Published:** 2022-07-25

**Authors:** Saskia Lehr, Felicitas Felber, Iliana Tantcheva-Poór, Christina Keßler, Rüdiger Eming, Alexander Nyström, Marta Rizzi, Dimitra Kiritsi

**Affiliations:** ^1^ Department of Dermatology, Medical Center University of Freiburg, Faculty of Medicine, University of Freiburg, Freiburg, Germany; ^2^ Department of Dermatology, University of Cologne, Cologne, Germany; ^3^ Department of Pediatrics, University Hospital Muenster, Muenster, Germany; ^4^ Department of Dermatology and Allergology, Philipps-Universität Marburg, Marburg, Germany; ^5^ Freiburg Institute for Advanced Studies (FRIAS), University of Freiburg, Freiburg, Germany; ^6^ Department of Rheumatology and Clinical Immunology, Medical Center University of Freiburg, Faculty of Medicine, University of Freiburg, Freiburg, Germany; ^7^ Center for Chronic Immunodeficiency, Medical Center University of Freiburg, Faculty of Medicine, University of Freiburg, Freiburg, Germany

**Keywords:** autoimmune bullous disease, skin blistering, collagen VII, BP180, PD-1 inhibitor, epidermolysis bullosa

## Abstract

Skin blistering disorders are associated with inherited defects in proteins involved in the dermal-epidermal adhesion or autoantibodies targeting those proteins. Although blistering in hereditary epidermolysis bullosa (EB) is pathogenetically linked to genetic deficiency of distinct proteins of the epidermis or the dermal-epidermal junction, circulating autoantibodies against these proteins have also been identified in EB patients. So far, autoantibodies have been considered bystanders in EB and active pathogenicity of them in EB has not been disclosed. In sera of a cohort of 258 EB patients, we found by ELISA in 22% of the patients autoantibodies against the bullous pemphigoid antigen BP180. The titers correlated negatively with collagen VII skin expression and positively with disease severity. Among those patients, we identified six (2.33%) with clinical features of an autoimmune bullous disorder (AIBD) and positive indirect immunofluorescence (IIF) staining. In literature, we found four more cases of EB patients developing disease-aggravating AIBD. Co-existence of these two rare skin disorders suggests that EB patients have a predisposition for the development of AIBD. Our work highlights that EB patients with increased itch or blister formation should be evaluated for additional AIBD and repeated screening for changes in autoantibody titers and skin-binding specificities is advised.

## Introduction

Skin blistering diseases result from deficiency of proteins essential for the stability of the dermal-epidermal junction (DEJ). Multiple causes exist, such as genetically induced deficiency or autoantibodies targeting these proteins (1). Hereditary epidermolysis bullosa (EB) is a group of disorders, defined by mechanically induced skin blistering (2). In autoimmune bullous diseases (AIBD) autoantibodies against adhesion proteins of the skin, here collectively called “anti-skin autoantibodies”, cause skin fragility, typically accompanied by itch (3). Anti-skin autoantibodies have been found in EB, but they are considered a secondary event, as they appear to not bind to the respective skin structures (4–6). Here, we investigated sera of 258 patients with EB simplex (EBS), junctional (JEB) or dystrophic (DEB) by ELISA for autoantibodies against BP180, the antigen in the most common AIBD, bullous pemphigoid (BP) (3).

## Materials and methods

In this retrospective analysis, 258 patients with molecularly-confirmed hereditary epidermolysis bullosa were included. Sera were collected from all patients during routine controls and commercial BP180 ELISAs were performed. Six patients presented with clinical features of autoimmune blistering disease. Thus, their sera were additionally used to perform indirect immunofluorescence (IIF) on human salt-split skin, western-blots and commercial BP230 - and collagen VII ELISAs. Serum from one patient was further analyzed for desmoglein 1 and 3 and desmocollin 1, 2 and 3. From one patient a skin biopsy was taken from perilesional skin to perform direct immunofluorescence (DIF). DIF, IIF, commercial BP180 NC16A ELISAs (MBL, Nagoya, Japan), BP230 (MBL, Nagoya, Japan), collagen VII ELISAs (Euroimmun, Lübeck) and desmoglein 1 and desmoglein 3 ELISAs (MBL, Nagoya, Japan) were performed in the Department of Dermatology, University of Freiburg. Specialized anti-desmocollin 1, 2 and 3 IgA and desmoglein 1 and 3 IgA immunoblots were performed in the Department of Dermatology and Allergology, Philipps-Universität Marburg as previously described by Müller et al. (7). Patient material was collected and analyzed in accordance with the Declaration of Helsinki and after Ethics Approval (number 452/18). Patients gave informed written consent to the use for research and publication of their laboratory and clinical data, as well as clinical images.

## Results

**Figure 1:**
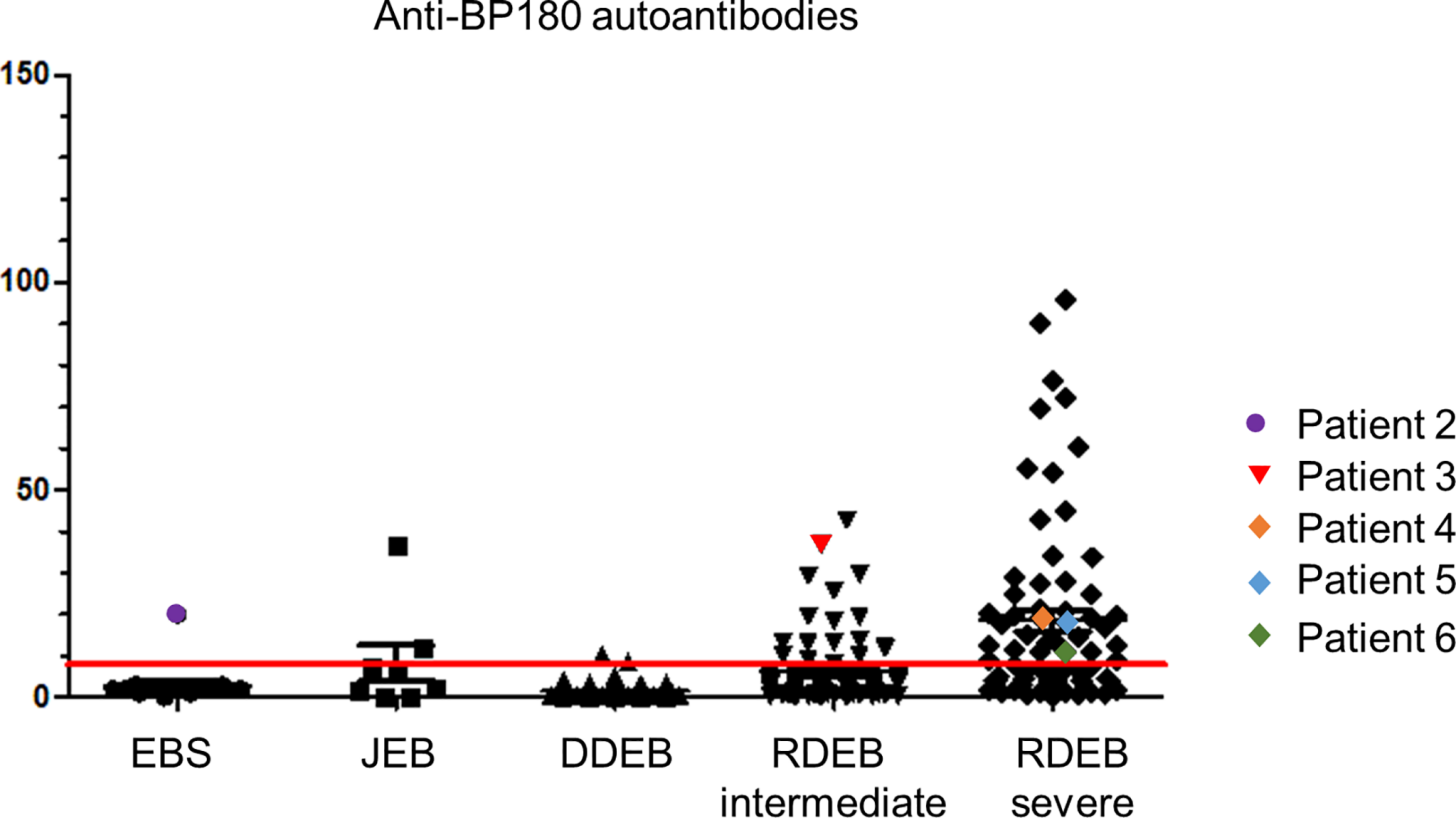
Anti-BP180 autoantibody titers of the patients in our EB-cohort. Anti-BP180 autoantibodies were detected by BP180 NC16A ELISA in patient sera. The red line indicates the upper limit norm level of 9 U/ml. EBS, EB simplex; JEB, junctional EB; DDEB, dominant dystrophic EB; RDEB, recessive dystrophic EB.

Around 5.3% (1/19) of EBS, 25.0% (2/8) of JEB and 23.4% (54/231) of DEB patients had high BP180 autoantibody titers, corresponding to those in BP. The titers correlated with EB severity, with 52,8% (38/72) of patients with severe recessive DEB (sRDEB) having BP180 titers above the threshold (**Figure 1**). Notably, in six patients with different EB subtypes, the BP180 autoantibodies correlated with clinical and serological manifestations of AIBD. In two cases, the disease developed after PD-1 (programmed cell death protein 1) inhibitor treatment (PD1i) for a metastasized or inoperable squamous cell carcinoma (SCC). PD1i are known to cause immune-related adverse events, which can actually manifest as AIBD (8). All 6 patients showed deterioration of the skin condition, manifesting as worsening of blister formation and/or itch and positive IIF, suggesting AIBD.

A thorough literature review identified four additional cases, in which EB patients did also develop AIBD (**Table 1** patient 7-10). Below, the patients of our EB-cohort with clinical features of AIBD and patients with EB and AIBD from the literature are briefly described; additional information is available in **Table 1**.

**Table 1:** Patients with EB and AIBD described in this study and in the literature.

Pat. No	Genetically confirmed EB type	Protein expression level (if available) or mutation	AIBD	DIF	IIF	Autoantibodies detected per ELISA	Treatment	Treatment response	Reference
1	EBS severe	*KRT5*: heterozygous c. 1429G > T/p.E477*, published in 9	BP	linear deposition of IgG at the DEJ	ss-IF (IgG): Staining on the epidermal side of the split	**BP180 IgG** (immunoblot)	initially topical treatment with high potency steroids and systemic treatment with doxycycline 200 mg/day and nicotinamide 400 mg/day, then change to dapsone 50 mg 2x/d	improvement under dapsone, but still itch and blisters	this study
2	EBS with plectin deficiency	plectin negative	BP	*NA*	ss-IF (IgG): Staining on the epidermal side of the split	**BP180 IgG (20 U/ml)**	topical treatment with medium potency steroids	itching and blister formation decreased significantly	this study
3	RDEB intermediate	collagen VII reduced	BP	*NA*	ss-IF (IgG): Staining on the epidermal side of the split	**BP180 IgG (37 U/ml)** **BP230 IgG (29 U/ml)** Col VII IgG (ratio 1.00)	topical treatment with medium potency steroids	*loss of follow-up*	this study
4	sRDEB	collagen VII negative	BP	negative (biopsy taken after steroid treatment)	ss-IF (IgG): Staining on the epidermal side of the split	**BP180 IgG (19 U/ml)** **BP230 IgG (20 U/ml)**	systemic treatment with prednisolone (starting dose 1 mg/kg bodyweight/day) followed by slow tapering and doxycycline 100 mg/day	itching and blister formation decreased, but flaired up during steroid tapering	this study
5	sRDEB	collagen VII negative	BP, PD1i associated	*NA*	ss-IF (IgG): Staining on the epidermal side of the split	**BP180 IgG (18 U/ml)** **BP230 IgG (14 U/ml)** Col VII IgG (ratio 1.03)	topical treatment with medium potency steroids	the already mild symptoms improved; PDi therapy was continued	this study
6	sRDEB	collagen VII strongly reduced	linear IgA bullous dermatosis with epitope spreading, PD1i associated	*NA*	ss-IF (IgA): Staining on the epidermal side of the split (IgG negative); IF on monkey esophagus (IgA): Intercellular staining (IgG negative)	**BP230 IgA** Col VII IgA **Desmocollin 2 IgA** **Desmocollin 3 IgA** **Desmoglein 3 IgA** Desmoglein 1 IgG (31 U/ml) Desmoglein 3 IgG (17 U/ml) BP180 IgG (10 U/ml) BP230 IgG (15 U/ml) Col VII IgG (ratio 1,52)	no specific therapy; PD1i treatment was stopped due to insufficient tumor response	itching decreased significantly a few weeks after discontinuation of PD1i treatment	this study
7	JEB intermediate (clinical diagnosis)	collagen XVII reduced	BP	linear deposition of IgG and C3 at the DEJ	ss-IF (IgG): staining on the epidermal side of the split	**BP180 IgG**	systemic treatment with prednisolone (starting dose 60 mg/day) and mycophenolate mofetil 1 g twice daily	acquired blistering was resolved (patient died a few month later from a brain tumor)	(10)
8	JEB generalized intermediate	laminin 332 reduced	BP	linear deposition of IgG and C3 at the DEJ	ss-IF (IgG): staining on the epidermal side of the split	**BP180 IgG**	initial systemic treatment with prednisolone (starting dose 40 mg/day), doxycycline 200 mg/day and nicotinamide 750 mg/day, then change to systemic and topical steroids and dapsone 50 mg/d for 2 month	clear improvement of BP after dapsone and systemic steroids (but not resolved)	(11)
9	RDEB “nails only”	collagen VII normal	EB acquisita	linear deposition of IgG at the DEJ	ss-IF (IgG): staining on the dermal side of the split	**ColVII IgG** BP180 IgG BP230 IgG Laminin α3 chain IgG Laminin β3 chain IgG	sequential systemic treatment with prednisone (1 mg/kg/day) and colchicine (2 mg/day)	minimal clinical improvement, thereafter any therapy was refused by the patient	(12)
10	DDEB “mild”	collagen VII expression slightly reduced	EB acquisita	linear deposition of IgG and C3 at the DEJ	ss-IF (IgG): staining on the dermal side of the split	**ColVII IgG**	systemic treatment with prednisolone 0,5 mg/kg bodyweight/day	treatment efficacy was limited; the patient died 3 years after diagnosis of EBA	(13)

The autoantibodies, which we consider AIBD-causing are depicted in bold. The upper limit norms of antibody-titers from this study are the following BP180 = 9 U/ml, BP230 = 9 U/ml, desmoglein 1 = 14 U/ml, desmoglein 3 = 7 U/ml, collagen VII= ratio 1.00. Protein expression levels in patients 2-6 were evaluated by immunofluorescence stainings with the respective specific antibodies. AIBD, autoimmune bullous diseases; BP, bullous pemphigoid; ColVII, collagen VII; DEB, dystrophic epidermolysis bullosa; DEJ, dermal-epidermal junction; DIF, direct immunofluorescence; EB, epidermolysis bullosa; EBS, EB simplex; IIF, indirect immunofluorescence; JEB, junctional EB; NA, not available; ss-IIF, indirect immunofluorescence on human salt-split skin; sRDEB, severe recessive DEB; PD1i, programmed cell death protein 1 inhibitor.

### Spontaneous AIBD in the cohort of this study

**Figure 2:**
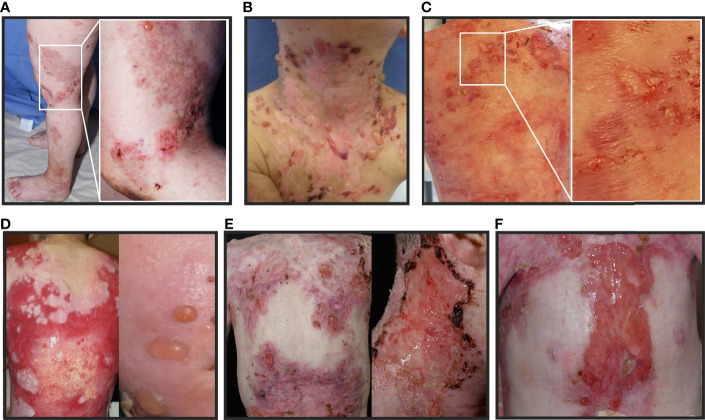
Clinical pictures of the patients with EB and AIBD described in this study: **(A–F)**: Patients present with tense blisters, erosions and ulcerations simultaneously, which are characteristic for both, EB and AIBD. Therefore, conclusive clinical differentiation is challenging. **(A)** patient 1, **(B)** patient 2, **(C)** patient 3, **(D)** patient 4, **(E)** patient 5 under therapy with programmed cell death protein 1 inhibitor (PD1i) and **(F)** patient 6 under therapy with PD1i.

**Figure 3:**
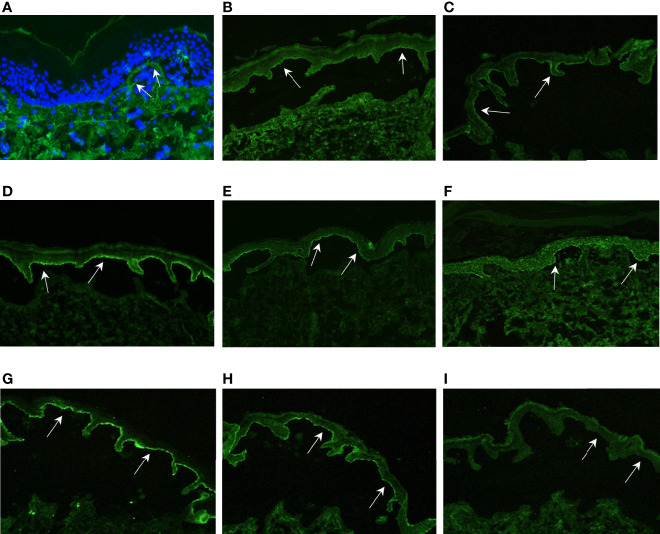
Immunofluorescence results. **(A)** DIF of patient 1 shows discrete linear deposition of IgG at the DEJ (white arrows); **(B–F)**: ss-IIF show IgG staining on the epidermal side of the split, indicated by white arrows **(B)** = Pat. 1, **(C)**= Pat. 2, **(D)** = Pat. 3, **(E)** = Pat. 4, **(F)** = Pat. 5); **(G)** ss-IIF IgA shows IgA staining on the epidermal side of the split (white arrows)*;* **(H)** ss-IIF IgG positive control with IgG staining on the epidermal side of the split, indicated by white arrows; **(I)** ss-IIF IgG negative control without IgG staining on the epidermal side of the split, indicated by white arrows. The IgA positive control was comparable to the staining in **(H)** (not shown).

The 61-year-old patient 1 with severe EBS caused by a heterozygous keratin 5 mutation (9) suddenly suffered from intensified itch and formation of tense blisters on legs, arms and trunk in contrast to her usual acral predilection sites (**Figure 2A**). BP diagnosis was confirmed by histopathology, direct immunofluorescence (DIF) (**Figure 3A**) and indirect immunofluorescence on salt-split skin (ss-IIF) (**Figure 3B**) as well as detection of anti-BP180 autoantibodies per immunoblot.

The 4-year-old patient 2 with EBS presented with increased itching and worsened skin fragility with up to 20 new tense blisters daily (**Figure 2B**). Ss-IIF (**Figure 3C**) and elevated BP180 autoantibody titers were consistent with BP diagnosis.

Patient 3 was a 9-year-old boy with intermediate RDEB. He was already severely affected but experienced a sudden intensification of itch and development of tense blisters, mainly at the trunk (**Figure 2C**). BP diagnosis was confirmed by ss-IIF (**Figure 3D**) and elevated anti-BP180/BP230 autoantibody titers.

The 14-year-old patient 4, diagnosed with sRDEB, suffered from increasing itch, increased wound burden and formation of tense blisters in the face (**Figure 2D**). Ss-IIF (**Figure 3E**) and high BP180 and BP230 autoantibody titers revealed the diagnosis of BP.

### PD1i-therapy associated AIBD in the cohort of this study

The 39-year-old patient 5 with sRDEB received PD1i therapy for an inoperable SCC at his right hand. His wound burden increased steadily over time (**Figure 2E**). The patient denied intensified itch, but treatment with cannabinoids for cancer-related pain was introduced simultaneously. Ss-IIF (**Figure 3F**) and elevated anti-BP180/BP230 autoantibody titers were compatible with BP.

The 26-year-old patient 6 with sRDEB had been diagnosed with multiple SCCs in the past and received PD1i treatment for his lymph node metastases. A few weeks after PD1i initiation, he suffered from a sudden increase of wound burden (**Figure 2F**) and severe itch. IgG autoantibodies against BP180, BP230 and collagen VII were detected by ELISA, there was no IgG deposition in IIF. However, ss-IIF revealed IgA at the blister roof (**Figure 3G**) and IIF on monkey esophagus showed intercellular IgA deposition. Immunoblotting with keratinocyte extracts revealed IgA-autoantibodies against collagen VII and BP230. In addition, IgA-autoantibodies against desmoglein 3, desmocollin 2 and desmocollin 3 were detected. Occurrence of autoreactive IgA antibodies binding to DEJ proteins are compatible with linear IgA bullous dermatosis, and the cell surface pattern on monkey esophagus indicates epitope spreading. SS-IIF stainings from all patients were comparable to ss-IIF positive controls with IgG staining (**Figure 3H**) or IgA staining (not shown) on the epidermal site of the split and clearly distinguishable from negative controls without IgG staining on the epidermal site of the spilt (**Figure 3I**).

### Patients with EB and AIBD described in the literature

Perez et al. (10) describe a 56-year-old patient (**Table 1**, Patient 7) with a clinically diagnosed intermediate junctional epidermolysis bullosa, who suddenly developed spontaneous blisters, in addition to mechanically inducible blisters and nail dystrophy, which he had his lifelong. DIF, ss-IIF and the detection of autoantibodies against BP180 by ELISA were compatible with the diagnosis of BP.

Fania et al. (11) reported a 32-year-old patient (**Table 1**, Patient 8) with generalized intermediate junctional epidermolysis bullosa and sudden development of a rash with vesiculobullous and excoriated lesions, accompanied by severe itch. The diagnosis of BP was confirmed by DIF, positive ss-IIF and detection of BP180 autoantibodies by western blot and ELISA.

Guerra et al. (12) describe a 32-year-old patient (**Table 1**, Patient 9) with “nails only” RDEB, who presented with mechanically induced blisters and oral erosions at the age of 26. DIF, ss-IIF and the detection of collagen VII autoantibodies confirmed the diagnosis of epidermolysis bullosa acquisita (EBA).

Hayashi et al. (13) reported a 63-year-old patient (**Table 1**, Patient 10) with mild DDEB and sudden development of blisters, erosions and scars all over her body, including the oral mucosa. The diagnosis of EBA was approved by DIF, ss-IIF and anti-collagen VII autoantibodies detected by western blot.

## Discussion

Occurrence of circulating anti-skin autoantibodies in EB patients appears to be frequent, but usually DIF and IIF are negative (4-6, 14, 15). So far, the association of inherited EB and AIBD had been reported in four (**Table 1**, patients 7-10) and we describe six further cases (**Table 1**, patients 1-6).

With 2.33%, the AIBD prevalence in our large EB cohort was considerably higher than the estimated AIBD prevalence of 0.05% in the general population of Germany (16). The rarity of these diseases and the BP onset at remarkably young age in 6/10 cases, considering the mean age of 80 years for BP onset in the general population (16), suggests that EB patients have a predisposition for the development of AIBD.

It remains elusive, how autoantibody formation occurs in EB, and why only in some patients (1). Molecular mimicry between foreign (e.g. bacterial or viral antigens) and self-proteins could trigger autoantibody formation (17). In EB, chronic impairment of skin barrier results in constant exposure to bacteria, making molecular mimicry likely to occur (4). Alternatively, genetic variants in skin proteins could enhance the immunogenicity of the self antigens, favoring the activation of self-reactive B cells (12), as we recently also showed in patients with EB acquisita (18). Genetically altered DEJ proteins could also contribute to autoimmunity, by causing endoplasmic reticulum stress and activation of the unfolded protein response (11). Existence of several anti-skin autoantibodies in one EB patient as already reported (4) and shown in four of our patients, could be explained by epitope spreading (19). Development of secondary epitopes in AIBD could be provoked by release of cryptic epitopes due to chronic tissue damage, caused by the response against the dominant epitope (19). In EB, this could be the consequence of tissue damage arisen by the genetic defect (4, 18).

All these mechanisms may contribute to generation of self-reactive antibodies, but they do not explain their inconsistent pathogenicity, with a clinical picture of AIBD developing only in selected patients. Factors determining pathogenicity of autoantibodies include antigen specificity, immunoglobulin isotype and the glycosylation/sialylation patterns of their Fc-regions (20). The development of AIBD in two sRDEB patients after PD1i treatment, which had non-pathogenic anti-skin autoantibodies before, is an indication that additional changes in the immune system, including T cell activation, are necessary to induce the final break of tolerance.

Our study is limited by the lack of an age- and sex matched healthy control group. However, in a large cohort of 7036 healthy individuals, Prüßmann et al. found anti-skin autoantibodies in only 0.88%, with anti-BP180 autoantibodies being the most common with 0.52% (21). Thus, the prevalence of anti-BP180 autoantibodies in our EB cohort was 42 times higher than in the general population. Another weakness is that further evaluation of the antibodies’ pathogenicity was only performed if clinical criteria (worsening of blister formation, intensified itch) pointed towards an AIBD. Since IIF staining was not performed systematically for all patients, we cannot exclude that even more patients of our cohort actually have binding anti-skin antibodies. Finally, our study is restricted by the lack of DIF in most patients, who refused having biopsies of their inflamed and wounded skin. Thus, AIBD diagnosis was based on clinical and serological criteria.

This work highlights that if an EB patient develops symptoms such as increased itch and blister formation, involvement of autoantibodies to these disease manifestations should be considered. AIBD under PD1i can be variable and very mild (8), thus clinical diagnosis is difficult. If AIBD is suspected in an EB patient, IIF or DIF should be performed to test skin-binding specificity of the detected autoantibodies. Treatment is challenging, since immunosuppression is contra-indicated in JEB and DEB with risk for development of highly aggressive SCCs (22), but a better understanding of B cell involvement could provide new therapeutic targets.

## Data Availability Statement

The raw data supporting the conclusions of this article will be made available by the authors, without undue reservation.

## Ethics Statement

The studies involving human participants were reviewed and approved by the Ethics Committee of the Albert-Ludwigs-University Freiburg (Ethics Approval number 452/18). Written informed consent to participate in this study was provided by the participants’ legal guardian/next of kin. Written informed consent was obtained from the individual(s), and minor(s)’ legal guardian/next of kin, for the publication of any potentially identifiable images or data included in this article.

## Author Contributions

Conceptualization: DK; Data curation: SL; Formal analysis: SL; Funding acquisition: DK and AN; Investigation: SL, FF, IT-P, CK, and RE; Methodology: DK and SL; Project administration: DK and AN; Resources: DK and AN; Supervision: DK, MR, and AN; Validation: IT-P, CK, and AN; Visualization: SL; Writing – original draft: SL and DK; Writing – review & editing: FF, IT-P, CK, RE, AN, and MR. All authors contributed to the article and approved the submitted version.

## Funding

SL and DK are funded by the Deutsche Forschungsgemeinschaft (DFG, German Research Foundation) - CRC1160/2 - B03(N), Medical Center - University of Freiburg, and Faculty of Medicine, University of Freiburg. This work was further supported by the Berta-Ottenstein Advanced Clinician Scientist Programme of the University of Freiburg to DK and by the German Research Foundation (DFG) through KI1795/2-1 and the CRC-1479 – Project ID: 441891347 to DK.

## Acknowledgments

We thank the patients and acknowledge the work of our fellow physicians in the Epidermolysis bullosa Center Freiburg, especially Prof. Bruckner-Tuderman for the valuable suggestions. We thank Kaethe Thoma and Annegret Bedorf for the technical assistance.

## Conflict of Interest

The authors declare that the research was conducted in the absence of any commercial or financial relationships that could be construed as a potential conflict of interest.

## Publisher’s Note

All claims expressed in this article are solely those of the authors and do not necessarily represent those of their affiliated organizations, or those of the publisher, the editors and the reviewers. Any product that may be evaluated in this article, or claim that may be made by its manufacturer, is not guaranteed or endorsed by the publisher.

## Abbreviations

AIBD, autoimmune bullous diseases; BP, bullous pemphigoid; DEB, dystrophic epidermolysis bullosa; DEJ, dermal-epidermal junction; DIF, direct immunofluorescence; EB, epidermolysis bullosa; EBS, epidermolysis bullosa simplex; IIF, indirect immunofluorescence; JEB, junctional epidermolysis bullosa; ss-IIF, indirect immunofluorescence on human salt-split skin; SCC, squamous cell carcinoma; sRDEB, severe recessive dystrophic epidermolysis bullosa; PD-1, programmed cell death protein 1; PD1i, programmed cell death protein 1 inhibitor.
